# Enhanced LSTM-based robotic agent for load forecasting in low-voltage distributed photovoltaic power distribution network

**DOI:** 10.3389/fnbot.2024.1431643

**Published:** 2024-07-11

**Authors:** Xudong Zhang, Junlong Wang, Jun Wang, Hao Wang, Lijun Lu

**Affiliations:** ^1^State Grid Hebei Electric Power Company, Shijiazhuang, China; ^2^Henan XJ Metering Co., Ltd, Xuchang, China

**Keywords:** distributed photovoltaic, power distribution network, load forecasting, deep learning, long short-term memory

## Abstract

To ensure the safe operation and dispatching control of a low-voltage distributed photovoltaic (PV) power distribution network (PDN), the load forecasting problem of the PDN is studied in this study. Based on deep learning technology, this paper proposes a robot-assisted load forecasting method for low-voltage distributed photovoltaic power distribution networks using enhanced long short-term memory (LSTM). This method employs the frequency domain decomposition (FDD) to obtain boundary points and incorporates a dense layer following the LSTM layer to better extract data features. The LSTM is used to predict low-frequency and high-frequency components separately, enabling the model to precisely capture the voltage variation patterns across different frequency components, thereby achieving high-precision voltage prediction. By verifying the historical operation data set of a low-voltage distributed PV-PDN in Guangdong Province, experimental results demonstrate that the proposed “FDD+LSTM” model outperforms both recurrent neural network and support vector machine models in terms of prediction accuracy on both time scales of 1 h and 4 h. Precisely forecast the voltage in different seasons and time scales, which has a certain value in promoting the development of the PDN and related technology industry chain.

## 1 Introduction

Load forecasting of the power distribution network (PDN) is an important link in safe operation and dispatching control. With the popularization and application of energy storage technology and the addition of new dispatchable resources such as electric vehicles, a large number of interruptible and bidirectional loads appear on the load side (Dairi et al., 2020; Razavi et al., 2020; Markovics and Mayer, 2022). These load's randomness and distributed access characteristics affect the power system regulation of the PDN. Active distribution network (ADN) uses the core technology of demand response to dynamically adjust the price of electricity and incentive policies and flexibly manage and control the original load demand of users. Furthermore, it actively guides users to participate in the optimization of power dispatching to enhance the synergy and complementarity of multiple loads. It not only considers users' satisfaction with electricity consumption but also improves the consumption ratio of distributed renewable energy (Hafiz et al., 2020; Mellit et al., 2021).

Proper planning and useful applications of load forecasting of the PDN require specific “predicting intervals”. According to the delivery cycle, load forecasting can be divided into ultra-short-term, short-term, medium-term, and long-term (Eom et al., 2020). Ultra-short-term forecasting is employed for real-time control, enabling rapid adjustments to generation and load to ensure the safe and stable operation of the power grid. Short-term forecasting is widely employed in the daily operations of the utility industry, facilitating dispatch of generation and transmission, optimizing grid resource allocation and enhancing grid operational efficiency. Medium-term forecasting is primarily utilized to forecast load variations over the next few months to a year, providing valuable insights for fuel procurement, maintenance planning and grid investment decisions. Long-term forecasting focuses on load growth trends over the next 1 to 20 years, employed to forecast the need for new power plants, grid planning and providing strategic guidance for power system development.

Load forecasting of the PDN is complex for engineers and academics, and remains an ongoing area of research. Moreover, the thorough exploration of load-side controllable resources to achieve optimal dispatch of the power system by the grid has emerged as a critical research priority for contemporary power utilities. Nowadays, it is more and more common for low-voltage PDNs to adopt distributed photovoltaic (PV) access. On this basis, considering the regularity of PV power generation, the problem of voltage fluctuation can be solved by predicting the voltage variation trend.

Accurate load forecasting plays a crucial role in optimizing the scheduling and management of power resources, effectively reducing operational costs and enhancing the overall efficiency of the power system. With the rapid development of deep learning-based robotic agent technology (Ma et al., 2023, 2024a), the application of deep learning in load forecasting has gained significant attention, particularly for approaches based on recurrent neural networks (RNN). Furthermore, deep learning techniques can handle complex nonlinear relationships and massive datasets, thereby improving the accuracy and reliability of predictions, which are paramount for the stable operation of the power grid. Deep learning models, however, demand substantial data and computational resources, while their hyperparameter tuning and training process necessitate specialized knowledge and expertise. The nonlinearity and time dependence of load data increase the complexity of predictions. As the data dimensions increase, deep learning models need to possess enhanced learning capabilities, thereby avoiding overfitting and performance degradation. While significant progress has been made in load forecasting techniques, several challenges remain that require further attention to enhance the accuracy and efficiency.

To address the specific scenario of load forecasting in low-voltage distributed photovoltaic power distribution networks, we customized a load forecasting model and employed a long short-term memory (LSTM) network architecture for forecasting. To enhance feature extraction, we placed a fully connected layer, denoted as dense layer, after the LSTM layer. Additionally, we integrated the frequency domain decomposition (FDD) method to obtain the amplitude and phase of each frequency component, and utilized LSTM to individually forecast low-frequency and high-frequency components, ultimately improving the model's accuracy. This study is expected to offer a new idea for the low-voltage distributed PV-PDN to meet the forecast. The contributions of this paper can be summarized as follows:

1) FDD-enhanced LSTM for load forecasting in PV-PDN: to address the load forecasting of low-voltage distributed PV-PDN, we propose a novel FDD-enhanced LSTM model. The proposed model outperforms conventional support vector machine (SVM) and RNN models, particularly in long-term forecasting scenarios. This method represents a significant advancement in the application of deep learning techniques in the distribution network domain, providing a novel approach to enhance grid reliability and operational efficiency.
2) A new benchmark for load forecasting in PV systems: the integration of FDD and LSTM networks has revolutionized load forecasting in low-voltage distributed PV systems, establishing a new benchmark for forecasting methodologies in distributed PV systems.
3) Comparative analysis of FDD-enhanced LSTM for load forecasting: to objectively evaluate the performance of the enhanced LSTM model in complex low-voltage distributed PV forecasting scenarios, we conducted a comprehensive comparative analysis of the mean absolute error (MAE) across different time scales. The results demonstrate the model's superior performance and reliability in complex voltage forecasting environments.

The rest of the paper is organized as follows: Section 2 reviews the related work of load forecasting and scene image monitoring analysis. Section 3 describes the proposed methods in detail. Section 4 reports the experimental result and analysis. Section 5 represents the conclusion and future work.

## 2 Related work

Low-voltage load forecasting is an intelligent technique that utilizes historical load data, weather information and socioeconomic factors to forecast future low-voltage load levels. This technique possesses extensive application value in power grid scheduling, grid planning and electricity pricing.

Statistical and time series methods are widely employed techniques for short-term load forecasting, with linear models being the most prevalent approach. Linear models typically employ linear parameters. Litjens et al. (2018) have utilized some of the simplest linear models, including seasonal persistence models and simple average models, often in conjunction with meteorological data. Borges et al. employed linear models with varying feature subsets for short-term load forecasting and missing data imputation in substation data (Borges et al., 2020). Their model utilized historical load data, meteorological data and neighboring substation data. While standard linear regression has proven successful in demand forecasting for all levels of low-voltage networks, nonlinear regression models have also gained attention due to their inherent flexibility. Hayes et al. employed a nonlinear autoregressive exogenous (NARX) model for smart meter load forecasting and demonstrated its superior performance compared to traditional NARX models and neural network models (Hayes et al., 2014). Tsekouras et al. (2007) employed nonlinear multiple regression, selecting a model based on testing various combinations of nonlinear functions for mid-term load forecasting. Nonlinear models, despite their wide applicability, are susceptible to overfitting issues.

Among time series forecasting models, ARIMA stands out due to its exceptional performance and has been widely adopted across various applications (Marinescu et al., 2013). Researchers have successfully integrated online ARIMA models into short-term forecasting of electricity systems in public school buildings (Lee et al., 2013). Leveraging historical load and temperature data, this model effectively captures energy efficiency, forecasts energy consumption and detects anomalies in energy usage. Furthermore, Espinoza et al. proposed a unified modeling framework based on periodic autoregressive models, enabling the effective integration of data from multiple entities to achieve load curve forecasting and clustering analysis (Espinoza et al., 2005).

With the continuous advancement of deep learning (Ma et al., 2021, 2024b; Li et al., 2023; Jin et al., 2024; Liufu et al., 2024), deep learning-based load forecasting has also gained widespread attention from researchers. Deep learning-based load forecasting methods, with their ability to capture complex data patterns and extract deep-level features, have gradually become a research hotspot in the field of power load forecasting and have achieved remarkable results. Shivam et al. (2021) discuss a predictive energy management strategy for residential PV-battery systems using RNN model, it has a deep inner hidden layer, which imitates the neural network inside humans to think like the human brain. Luo et al. (2021) enhance photovoltaic power generation forecasting by incorporating domain knowledge into deep learning models (Kim et al., 2020). The limitation of machine learning (ML) lies in the need for more learning ability for high-dimensional data. The purpose of representative learning is to simplify complex original data, remove invalid or redundant information from original data, and refine effective information to form features. The purpose of representative learning is to simplify complex raw data, remove redundant or invalid information from the data, and extract effective information to form features. In addition, SVM and LSTM have been widely used in load forecasting. Kabilan et al. (2021) and Feng et al. (2020) both employ machine learning models for short-term power prediction and quantifying daily global solar radiation, respectively, highlighting the potential of computational methods in optimizing and accurately forecasting solar energy production. Kim et al. (2020) focus on very-short-term photovoltaic forecasting for smart city energy management through multiscale LSTM-based deep learning.

In the realm of load forecasting, traditional methods have often faced limitations in capturing the intricate patterns and underlying relationships within complex electricity consumption data. To address these shortcomings, we propose a novel deep learning-based load forecasting framework that leverages the powerful capabilities of RNN and LSTM cells to effectively capture temporal dependencies.

## 3 Methods

### 3.1 Features of distributed PV-PDN

PV power generation is essentially a power technology that uses the photoelectric or photochemical effect of PV modules (semiconductor materials) to convert light energy directly into electric energy. Distributed PV power station usually refers to a power generation system with a small installed scale that uses distributed resources and is located near the user. Ordinarily, the power grid with a voltage level of <35 kV or lower is connected. The heart of a PV facility is solar panels. The semiconductor materials adopted for power generation principally cover polysilicon, monocrystalline silicon, amorphous silicon and cadmium telluride (Lopes et al., 2022). Solar panels are the core and most valuable part of a solar power system. Its role is to convert the radiant power of the sun into electrical energy, feed it into a storage battery or promote load operation. The function of the solar controller is to control the working state of the entire system and protect the battery from overcharge and discharge (Alipour et al., 2020; Korkmaz, 2021; Qadir et al., 2021). Qualified controllers should also have a temperature compensation function in places with large temperature differences.

**Figure 1:**
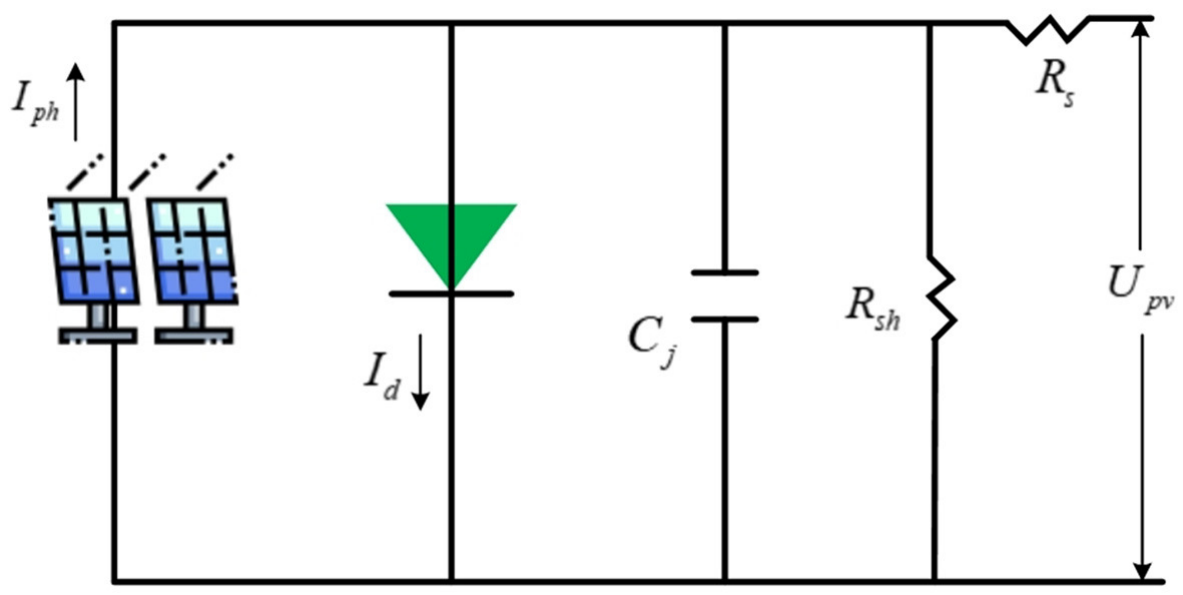
EC of PV cell.

The PV cell's equivalent circuit (EC) is shown in Figure 1. *I*_*ph*_ and *I*_*d*_ refer to the photo-generated and diode junction currents; *C*_*j*_ means the junction capacitance (negligible); *R*_*s*_ and *R*_*sh*_ stand for series and parallel resistors. Typically, distributed PV projects have a capacity of within a few kilowatts. Unlike centralized plants, the scale of PV plants has little effect on power generation efficiency. Therefore, its influence on the economy is also tiny, and the return on investment of small PV systems will not be lower than that of large ones.

**Figure 2:**
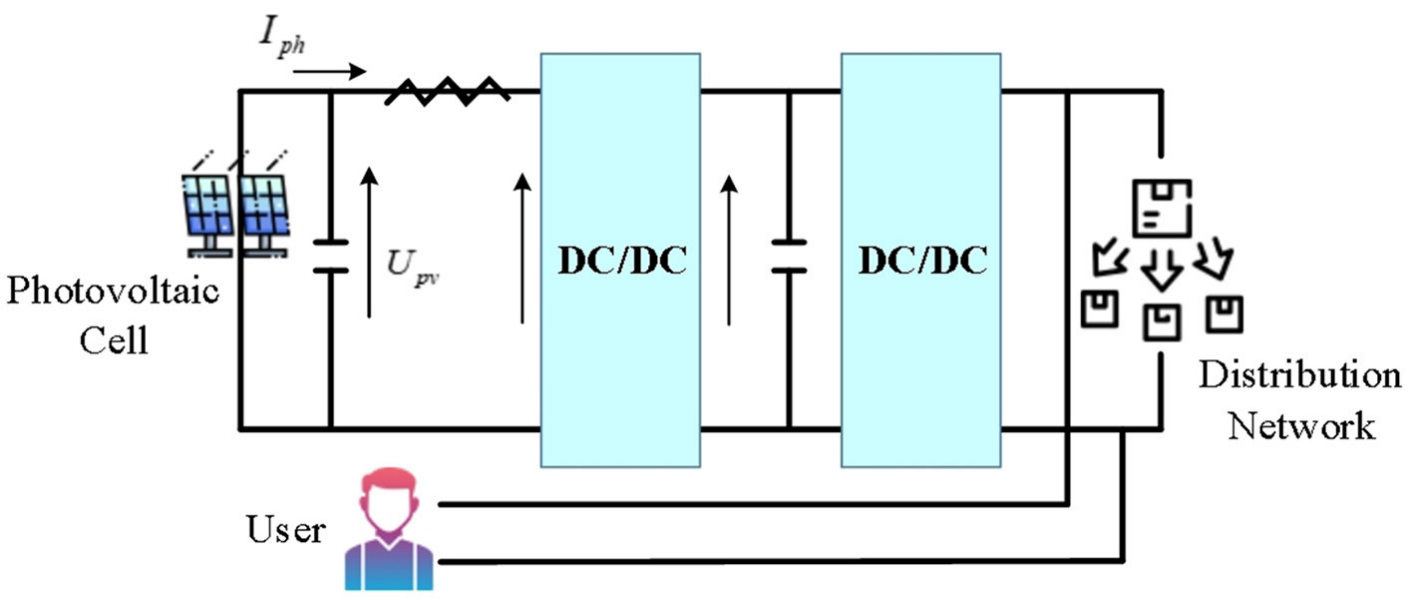
Grid-connected structure of household small distributed PV system.

Solar energy's direct output is generally 48 VDC, 24 VDC and 12 VDC. To power an appliance at 220 VAC, direct current (DC) generated by a solar power system needs to be converted into alternating current (AC). To avoid power backflow, it is necessary to configure an anti-flow device for alarm, and the inverter then adjusts its capacity according to the received signal. To connect the distributed PV system to the PDN, it first needs to output the PV cells through the DC/DC converter, then connected to the DC/AC inverter, and next connected to the external PDN. Taking a household small distributed PV system as an example, the typical grid-connected PV structure is displayed in Figure 2. The grid-connected access information acquisition system of small distributed PV power stations is applied to transmit the collected information to the monitoring platform and display it to users or power grid enterprises intuitively and clearly. This can provide grid enterprises with grid-connected data of PV power stations, eliminate the “blind adjustment” phenomenon of PV power generation, assist power grid operation analysis and decision-making, and promote the operation of the power grid safe and stable (Karimi et al., 2020; Ding et al., 2021; Khan et al., 2022).

**Figure 3:**
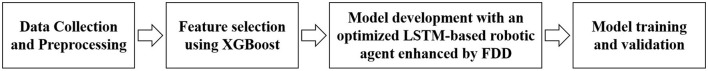
The overall diagram of the method in this study.

The overall diagram of the method in this study is shown as Figure 3. The method involves meticulous data collection and preprocessing to ensure high-quality inputs, followed by strategic feature selection via the XGBoost algorithm to optimize data relevancy. Then an advanced LSTM model is designed and refined, augmented with FDD, for enhanced predictive accuracy.

### 3.2 Voltage data preprocessing and feature selection

As a kind of clean energy, the high proportion of PV connected to a low-voltage PDN will bring huge power generation benefits. However, due to its own uncertainty, it may bring a series of problems to the stable and safe operation of the PDN, such as voltage over the limit, line overload and power quality reduction. Thus, it is essential to accurately evaluate the acceptance capacity of PV in a low-voltage PDN. More importantly, to further improve the benefits of PV power generation, it is urgent to improve the acceptance capacity of distributed PV based on accurate assessment (Rana and Rahman, 2020). Before voltage prediction of distributed PV-PDN, data mining and preprocessing should be carried out, ensuring that it is in a suitable form for analysis. This step involves removing outliers, handling missing values, and normalizing data, which helps reduce variability and improve the model's accuracy. It also includes feature selection and transformation to identify and utilize the most relevant information for forecasting, thereby enhancing the prediction model's effectiveness and efficiency.

The missing data is filled using the cubic spline interpolation fitting function *f*_θ_(*x*), and the equation for filling the value is Equation (1):


(1) $$D(t_{miss})=f_{\theta }\left(t_{miss}\right)$$

*t*_*miss*_ indicates the time point at which load data is missing.

For data satisfying normal distribution, standardized methods are used for dimensionless processing, with the specific equation as follows Equation (2):


(2) $$x^{*}=\frac{x-\bar{X}}{S}$$

*x* and x^*^ refer to the original and the processed feature data, respectively; $\bar{X}$ and *S* represent the mean and standard deviation of the feature, respectively.

DL model has advantages in capturing power voltage fluctuation in distributed PV voltage prediction due to their ability to model complex, nonlinear relationships within large datasets. They excel in identifying patterns and dependencies in temporal data, such as those found in voltage series, by leveraging multiple layers of processing. This capability allows DL models to provide more accurate and reliable forecasts of voltage fluctuations, which is essential for maintaining grid stability and optimizing energy distribution in distributed PV-PDN. At this time, dimensionless standardization of different power characteristics can significantly accelerate the optimization speed of the gradient descent algorithm. The maximum and minimum rescaling method of voltage and power is illustrated in Equations (3) and (4):


(3) $$v^{*}=\frac{v-v_{max}}{v_{max}-v_{min}}$$

(4) $$p^{*}=\frac{p-p_{max}}{p_{max}-p_{min}}$$

*v* & *p* and v^*^ & p^*^ represent time-series raw data and dimensionless data for voltage and power; *v*_max_ & *v*_min_ and *p*_max_ & *p*_min_ refer to the voltage data's and power data's maximum and minimum values, respectively.

Generally speaking, the power load is filled with data of similar size. Because the power load has a certain periodicity, it can be filled and replaced with similar load data of the same cycle. The power load has a regular periodicity, that is, the data of different periods at the same time should be very different. If the difference between the two data exceeds the threshold, the vertical method can be used for processing. For the PV system, the light intensity in winter is lower than that in summer, and the maximum light intensity is usually at noon, so the voltage will rise in this period. It can be seen that the time feature vector is very vital in the voltage prediction process, and it is a key factor in improving the prediction accuracy.

**Figure 4:**
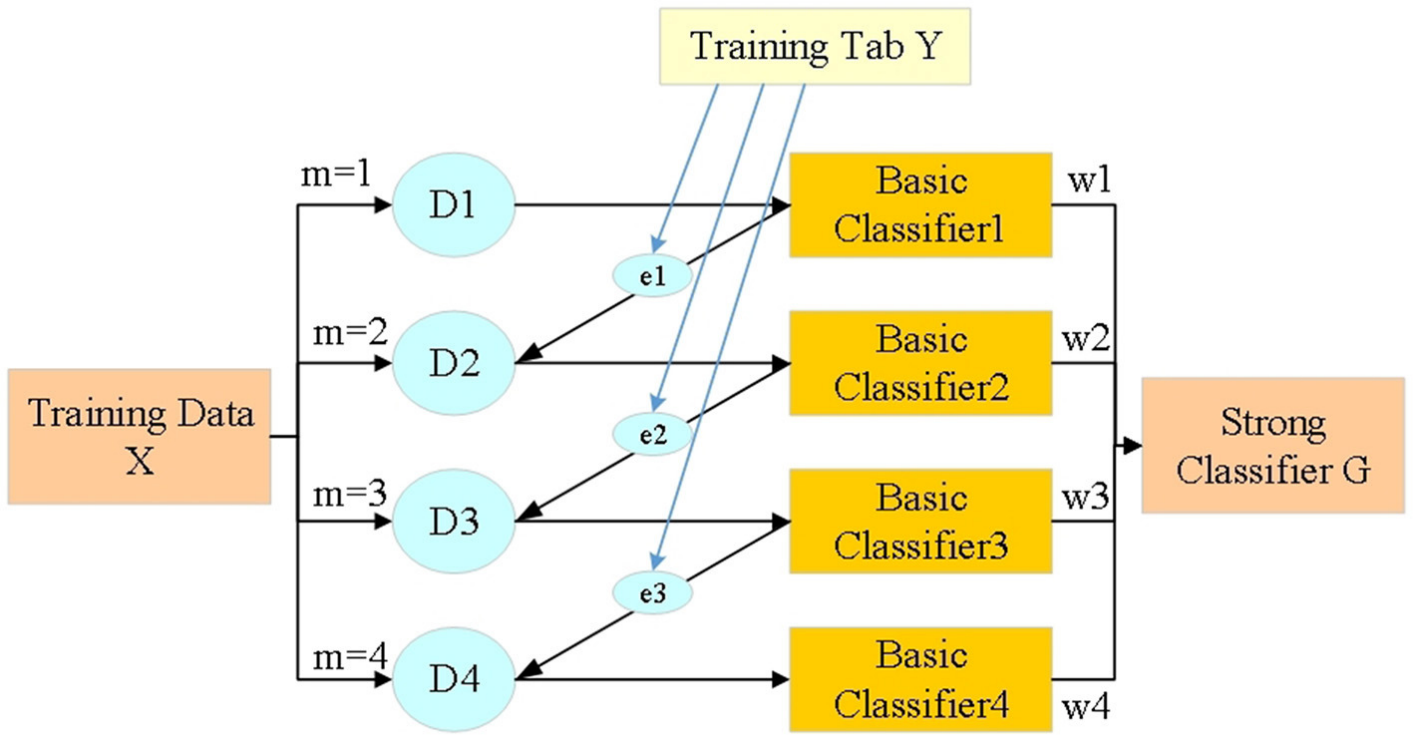
Training flow of XGBoost algorithm.

Considering various types of features in the voltage prediction process, this study will adopt the feature selection method based on the Extreme Gradient Boosting (XGBoost) algorithm (Bae et al., 2021), a method chosen for its efficiency and effectiveness in handling high-dimensional data. XGBoost is renowned for its ability to improve model performance by selecting the most relevant features, reducing noise and preventing overfitting. This approach aids in identifying the key predictors of voltage fluctuations in distributed PV systems, thus enhancing the predictive accuracy of the deep learning model. In the course of multiple iterations, the probability distribution (PD) of the training data used in the current iteration will be regulated based on the results of the previous iteration. That is to say, each sample of training data has a weight, which itself will be adjusted with iteration. As suggested in Figure 4, Dm is the training dataset's PD. In the first iteration, the classification error of basic classifier C1 is employed to adjust D2; In the second iteration, the base classifier C2 is used for iteration D3, and so on.

XGBoost is the use of multiple base learning. Each base learning is relatively simple. To prevent overfitting, the next learning is the result of learning the previous base learning. The loss function of XGBoost algorithm reads Equations (5) and (6):


(5) $$L=\sum_{i=1}^{n}l(y_{i},\;\hat{y_{i}})+\sum_{m=1}^{M}\Omega (b_{m})$$

(6) $$\Omega (b_{m})=\gamma T+\frac{1}{2}\lambda {\left\|w\right\|}^{2}$$

*n* refers to the number of samples; *y*_*i*_ and $\hat{y_{i}}$ represent the label value of the i-th sample and output value predicted by the model, respectively; *l* means the squared error function; Ω(*b*_*m*_) expressed a regularized term for the tree model. *T* displays the leaf nodes' quantities for a single tree model; *w* signifies the output vector of the leaf node; γ are λ parameters that control the weights of regularized terms.

After the model is initialized, it needs to carry out M-round cycle calculation, so the objective function *Obj*^(*t*)^ should be minimized during the t-round calculation Equations (7) and (8):


(7) $$Obj^{\left(t\right)}=\sum_{i=1}^{n}l(y_{i},\;{\hat{y_{i}}}^{\left(t-1\right)}+b_{t}(x_{i}))+\Omega (b_{t})+C$$

(8) $$C=\sum_{i=1}^{t-1}\left(b_{i}\right)$$

*b*_*t*_ represents tree model in the t-round training; ${\hat{y_{i}}}^{\left(t-1\right)}$ denotes the predicted output value of the model obtained from the previous round; Ω(*b*_*t*_) indicates the complexity of the tree model obtained in t-round; *C* is a constant.

When solving the objective function of a binary tree, it is necessary to know the first-order and second-order derivatives of the loss function, and on which leaf node the sample is located. It is also necessary to find the first and second derivatives of the sample at each leaf node to find the objective function. In this way, it is possible to decide whether to split the node and according to the characteristic values of which node to split.

The voltage, power of key nodes and time characteristics of prediction points in PDN are selected and taken as input feature vector *x* after series. When forecasting, the higher the prediction accuracy of 1 h ago, the higher the multiple time scales' prediction accuracy. The dimensionless node voltage and net power data of the complete PDN are obtained through data preprocessing. The voltage eigenvector *V*_*i*_ of the node, the net power eigenvector *P*_*i*_ and the corresponding label *y*_*i*_ are obtained as follows Equations (9–11):


(9) $$\begin{array}{l} V_{i}=\left[v_{i+t-H},...,v_{i+t-2},v_{i+t-1}\right] \end{array}$$

(10) $$\begin{array}{l} V_{i}=\left[p_{i+t-H},...,p_{i+t-2},p_{i+t-1}\right] \end{array}$$

(11) $$\begin{array}{l} y_{i}=v_{i+t} \end{array}$$

*H* represents the length of the sliding window, *V*_*i*_ and *V*_*i*_ are eigenvectors with dimension *H*.

The time variable of discretization is processed by unique thermal coding. Time eigenvector *T*_*i*_ is constructed to predict time points. Finally, the input feature vector *x*_*i*_ of the i-th sample can be expressed as Equation (12):


(12) $$\begin{array}{l} x_{i}=\left[V_{i},P_{i},T_{i}\right] \end{array}$$

*x*_*i*_ and *y*_*i*_ together constitute the training sample set of the XGBoost algorithm, which can be written as Equation (13):


(13) $$\begin{array}{l} {\left\{\left(x_{i},y_{i}\right)\right\}}_{i=1}^{n} \end{array}$$

Additionally, XGBoost suggests two ways to avoid overfitting. The first is Shrinkage, namely, the learning rate. In each tree iteration, each leaf node's weight is multiplied by a reduction coefficient. This way, the impact of each tree will not be too large, leaving more space for optimization for the trees below (Wang et al., 2017; Liu et al., 2022). Another way is Column Subsampling, which is similar to random forest selection for tree construction. There are two methods: (1) Random sampling by layer. Before splitting nodes of the same layer, some eigenvalues are randomly selected for traversal to calculate information gain (IG); (2) Some eigenvalues are randomly sampled before building a tree. Then the tree's all-node splits traverse these eigenvalues to compute IG.

The Mean Absolute Error (MAE) to validate the performance of prediction methods, which is an objective function used to measure the average absolute difference between predicted and true values in regression problems. It can measure the average error size between predicted values and true values, and has good robustness. The calculation formula for MAE is written as Equation (14):


(14) $$\begin{array}{l} MAE=\frac{1}{N}\overset{N}{\underset{i=1}{\sum }}|y_{i}-\hat{y_{i}}| \end{array}$$

*N* represents the number of samples, *y*_*i*_ is the true value, and $\hat{y_{i}}$ is the predicted value.

The smaller the value of MAE, the smaller the average difference between the predicted value and the true value, indicating higher accuracy of the prediction.

### 3.3 Load forecasting of distributed PV system based on FDD + LSTM

In the context of distributed photovoltaic systems, load forecasting necessitates a multifaceted analytical approach. Key is the scrutiny of historical data to discern patterns and trends. Employing statistical methods, such as time series analysis, facilitates the understanding of complex data interrelations. Moreover, the application of machine learning algorithms, including neural networks, is essential for improving prediction accuracy given the nonlinear nature of load data. Selecting pertinent features, particularly those influenced by weather and temporal factors, is critical. Additionally, integrating renewable energy sources, notably solar power, introduces unpredictability, demanding innovative, adaptable forecasting techniques to ensure consistent power distribution.

FDD refers to taking the Fourier transform (FT) of the signal to analyze it. FT is a mathematical equation that relates a signal sampled in space or time to the same signal sampled at frequency (Polo et al., 2023). In signal processing, FT can reveal a signal's vital characteristics (i.e., its frequency component). For a vector x containing n uniform sampling points, FT is defined as Equation (15):


(15) $$\begin{array}{l} y_{k+1}=\overset{n-1}{\underset{j=0}{\sum }}\omega^{jk}x_{j+1} \end{array}$$

ω is one of the *n* complex roots of unity; For *x* and *y*, indexes *j* and *k* range from 0 to *n*− 1.

**Figure 5:**
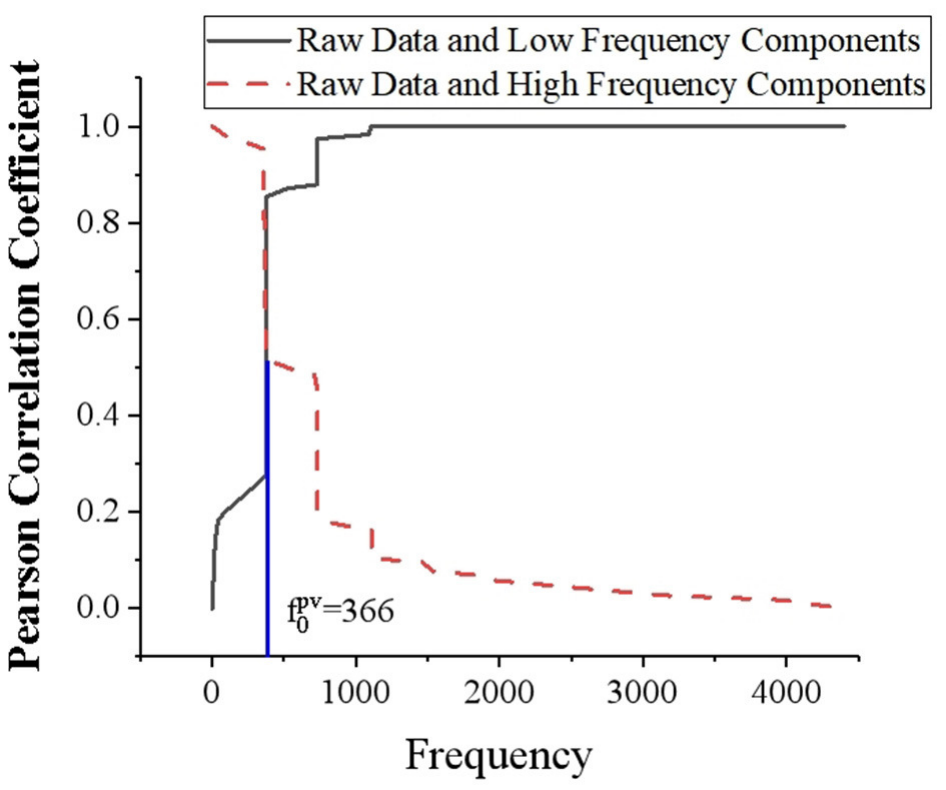
Correlation between high frequency and low-frequency components and PV power.

The Fourier analysis method is extended to aperiodic signals, and FT is introduced. When the period of a periodic signal increases infinitely, the frequency spectrum tends to become infinitely small and cannot be represented by the Fourier series. But from a physical point of view, the spectrum is still there. FT spectrum analysis divides PV power into load forecasting and high frequency components (Liu et al., 2020; Zang et al., 2020; Rai et al., 2021). The low-frequency component represents the conventional part of PV performance, which can be accurately predicted and indicates the trend characteristics. The high-frequency component exhibits the randomness of PV power and the fluctuation characteristics affected by weather and other factors, which is relatively difficult to predict. Figure 5 presents the correlation between low-frequency and high frequency components and PV power. When FDD is performed on PV power data, the more frequency is selected, the weaker the correlation between high frequency component and PV power is. However, the correlation between low-frequency component and photovoltaic power is stronger. Table 1 compares the predicted results of the two frequency components at different frequencies. The selection of frequency boundary points is based on frequency nodes with larger amplitude in the amplitude spectrum. It can be found that the core of frequency demarcation point selection is that the frequency selected by the low-frequency component should be as high as possible. Thereby, the low-frequency component accounts for more, and it is necessary to ensure that the frequency of the high frequency component is not too high, thus avoiding excessive difficulty in prediction.

**Table 1:** Predicted results of low-frequency and high frequency components at different frequencies.

**Com- ponent**	**R**					
	**366**	**732**	**1,098**	**1,463**	**1,830**	**2,196**
Low-frequency	0.985	0.986	0.987	0.988	0.989	0.990
High frequency	0.960	0.678	0.351	0.101	−2.175	−3.550

**Figure 6:**
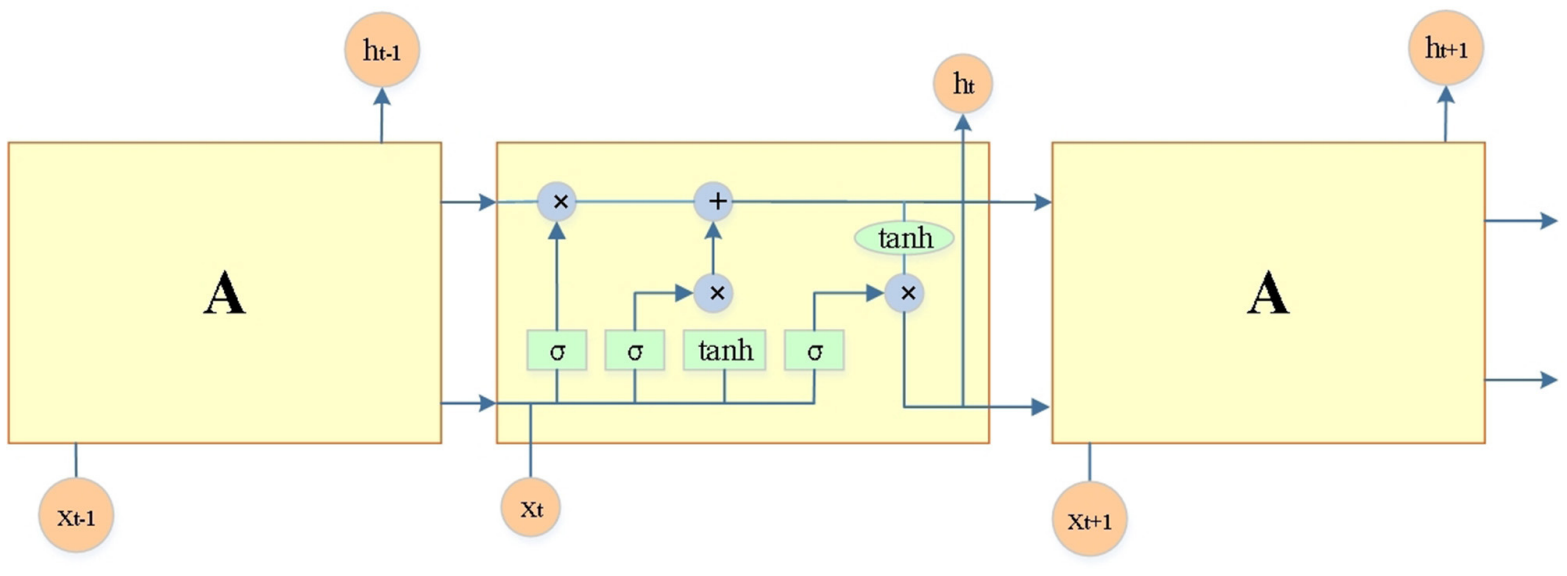
Basic structural unit of LSTM.

Convolutional networks can process images of different lengths and widths, and Recurrent Neural networks (RNN) have a recurrent function that can process data of different lengths and sequence types. However, due to the small range that RNN can utilize, it cannot handle the long sequence data well. The output that leads directly to a long sequence forgets the input that is farther away. LSTM is a special kind of RNN, a modified version of RNN, whose structure is plotted in Figure 6. The activation function is the sigmoid; tanh is the hyperbolic tangent function; ⊕ and ⊗ represent the addition and multiplication operations of vectors. The first layer of LSTM comprises a single-loop structure, which is determined by the dimensions and number of input data and loops, rather than the connection of multiple single-loop structures. LSTM cells contain input, forget, output and unit states (Akram et al., 2020; Zhang et al., 2020; Ahmad et al., 2022). The input gate determines how much network input data requires to be saved to the unit state at the current moment. The forget gate decides how many unit states need to be transferred from the last to the present moment. The output gate controls how much of the current unit state demands to be output to the present output value.

In the discussed PV-PDN voltage prediction model based on “FDD+LSTM”, to better extract data characteristics, a fully connected layer, namely Dense layer, is placed behind the LSTM layer. The specific voltage prediction process is as follows. (1) The prediction methodology employs XGBoost for feature subset selection, focusing on crucial elements like voltage, power characteristics and temporal variables. This step is pivotal in distilling the most relevant features from a vast dataset, thereby improving model efficiency and focus. The resulting feature vector *x* = [*V, P, T*] is a comprehensive aggregation of these elements, forming the LSTM input alongside the target training variable *y*_*i*_. (2) The backpropagation algorithm is utilized for model training, optimizing the network to reduce prediction errors and heighten voltage forecasting accuracy. This phase ensures in-depth learning from historical data, a critical aspect of the model's predictive capability. (3) Finally, the trained LSTM model, equipped with learned patterns, processes the input dataset for voltage prediction. The inclusion of the dense layer at this point is significant. It acts as a refinement stage, aligning LSTM outputs with expected voltage levels and synthesizing complex relationships. This addition enhances the model's accuracy and robustness in diverse operational scenarios within PV-PDNs.

Detailed procedure for load forecasting of distributed PV system based on FDD+LSTM:

(1) Data selection and preprocessing: historical operation data is carefully selected and subjected to data mining and preprocessing techniques. This includes handling outliers, addressing missing values and normalizing the data to ensure its suitability for analysis.
(2) Feature selection: to identify the most influential variables contributing to the prediction task, we employ the XGBoost algorithm for feature selection. This approach enables us to pinpoint key predictive factors such as voltage, power characteristics and time variables that significantly impact the target variable.
(3) Model training: to achieve accurate and reliable voltage predictions, we employ the proposed “FDD+LSTM” neural network architecture and train it using the backpropagation algorithm.
(4) Load prediction: to harness the predictive ability of the trained proposed “FDD+LSTM” model, we utilize it to process the input dataset for accurate voltage forecasting. To further enhance the model's ability to extract meaningful features from the data and improve prediction accuracy, we incorporate a dense layer into the network architecture.

## 4 Results and discussion

### 4.1 Data selection and example analysis

This study selects the historical operation data of a low-voltage distributed PV-PDN in Guangdong Province as the research object. The time range is operation data from March 2020 to March 2022. The data sampling interval of the meter under test is 1 h, and rolling prediction is adopted. The constructed input feature vectors *x*_*i*_ are: the vectors of voltage, power and time characteristics are 12, 12 and 35 dimensions, respectively, and a total of 16,275 data samples are constructed with *x*_*i*_ and label *y*_*i*_. An example analysis of the load forecasting model uses TensorFlow 14.0. The dropout layer is incorporated to prevent overfitting, followed by the connection to the output layer. The specific parameters of the “FDD+LSTM” prediction model are outlined in Table 2. The selected comparison algorithms are RNN, SVM, and LSTM to verify the validity of the prediction method proposed here.

**Table 2:** Specific parameters of LSTM prediction model.

**Model Layer**	**Hyper-parameter**	**Output tensor dimension**
Input layer	–	(None, input)
LSTM layer	Neurons in memory cells: 64	(None, 64)
Dense layer	Neurons: 128 Activation function: tanh	(None, 128)
Dropout layer	Drop rate: 0.05	(None, 128)
Output layer	Neurons: 1 Activation function: sigmoid	(None, 1)

### 4.2 Analysis of load forecasting results in distributed PV-PDN

**Figure 7:**
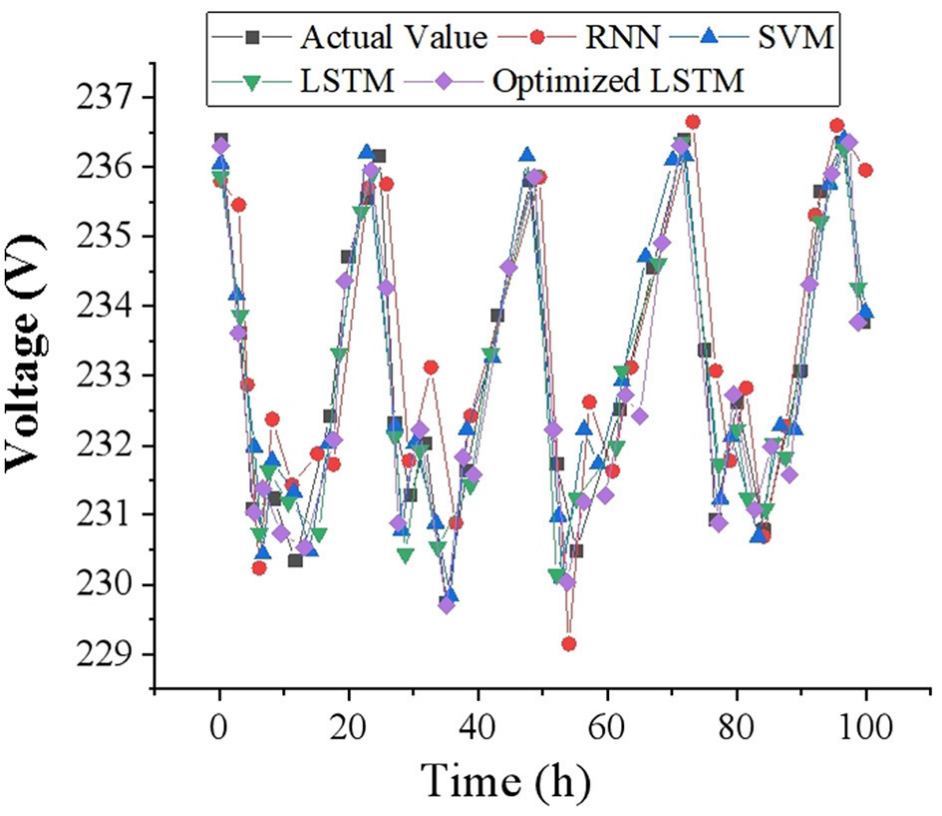
Voltage prediction results with a time scale of 1 h.

**Figure 8:**
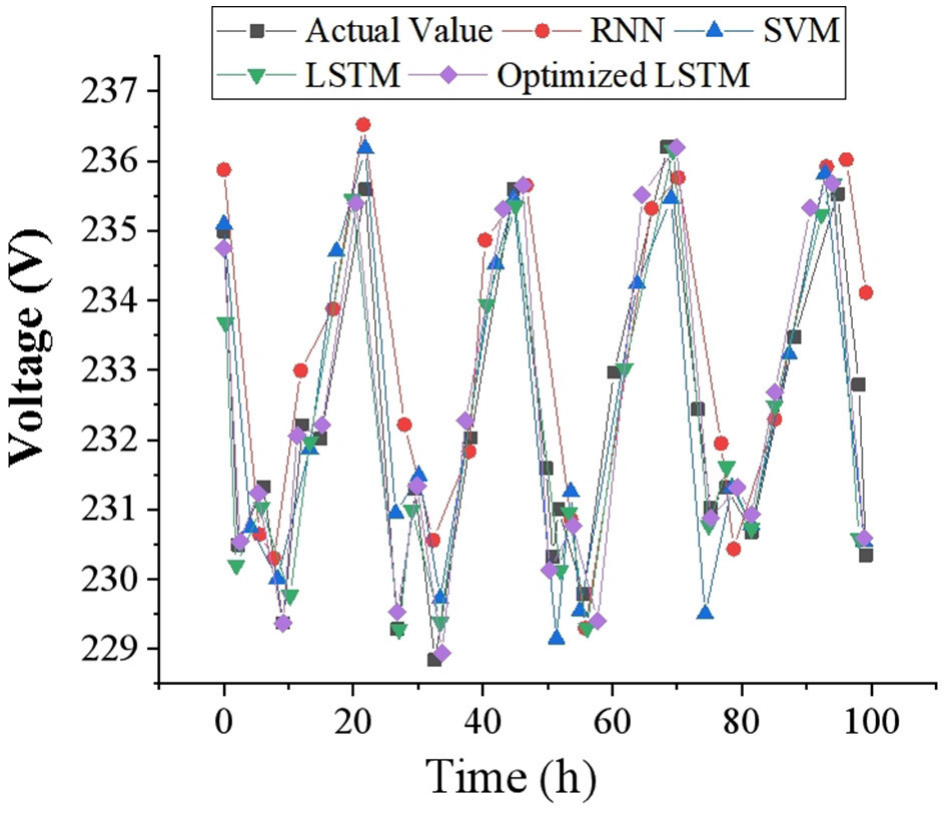
Voltage prediction outcomes with a time scale of 2 h.

**Figure 9:**
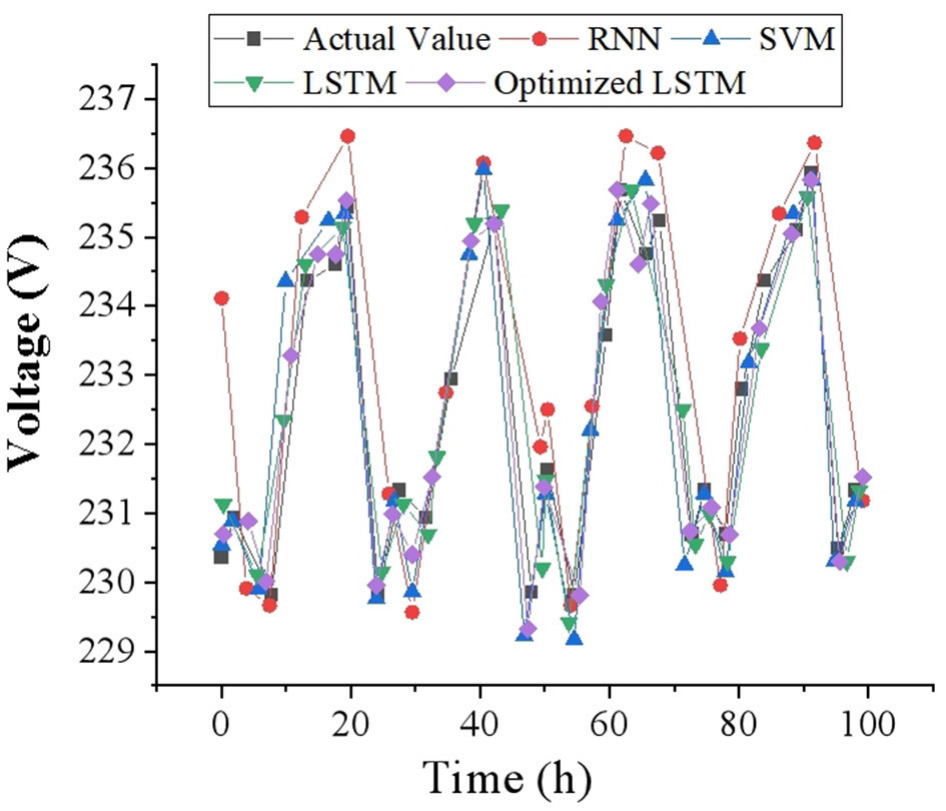
Voltage prediction results with a time scale of 4 h.

To intuitively reflect the accuracy of voltage prediction results, this study draws corresponding voltage prediction curves with 1, 2 and 4-h as time scales, and compares them with other prediction models. The voltage data of 100-time points is selected as a display in the test set, and the voltage prediction results at different time scales are demonstrated in Figures 7–9. It can be found that the FDD-enhanced LSTM model consistently aligns more closely with actual voltage values than SVM (Kabilan et al., 2021), RNN (Shivam et al., 2021) and LSTM (Feng et al., 2020) models, especially as the prediction time scale increases. Quantitatively, the LSTM model's MAE is significantly lower, at 0.4554 for a 1-h scale, compared to 0.535 and 1.012 for RNN and SVM, respectively. Even at a 4-h scale, the LSTM's MAE remains the lowest at 1.085. The superior forecasting precision of the optimized LSTM model can be attributed to its ability to effectively capture and learn from the temporal dependencies inherent in voltage data over time. Unlike SVM and RNN models, LSTM's architecture allows it to remember information for longer periods, making it particularly adept at handling the sequence prediction problems characteristic of voltage forecasting in distributed PV-PDNs. This is crucial for accurately predicting voltage fluctuations over different time scales, as it can account for both short-term and long-term patterns in the data. Additionally, the integration of FDD likely enhances the model's capability to deal with the non-linear and complex nature of the voltage signals, further improving prediction accuracy.

### 4.3 Performance evaluation of load forecasting model under different seasons

**Figure 10:**
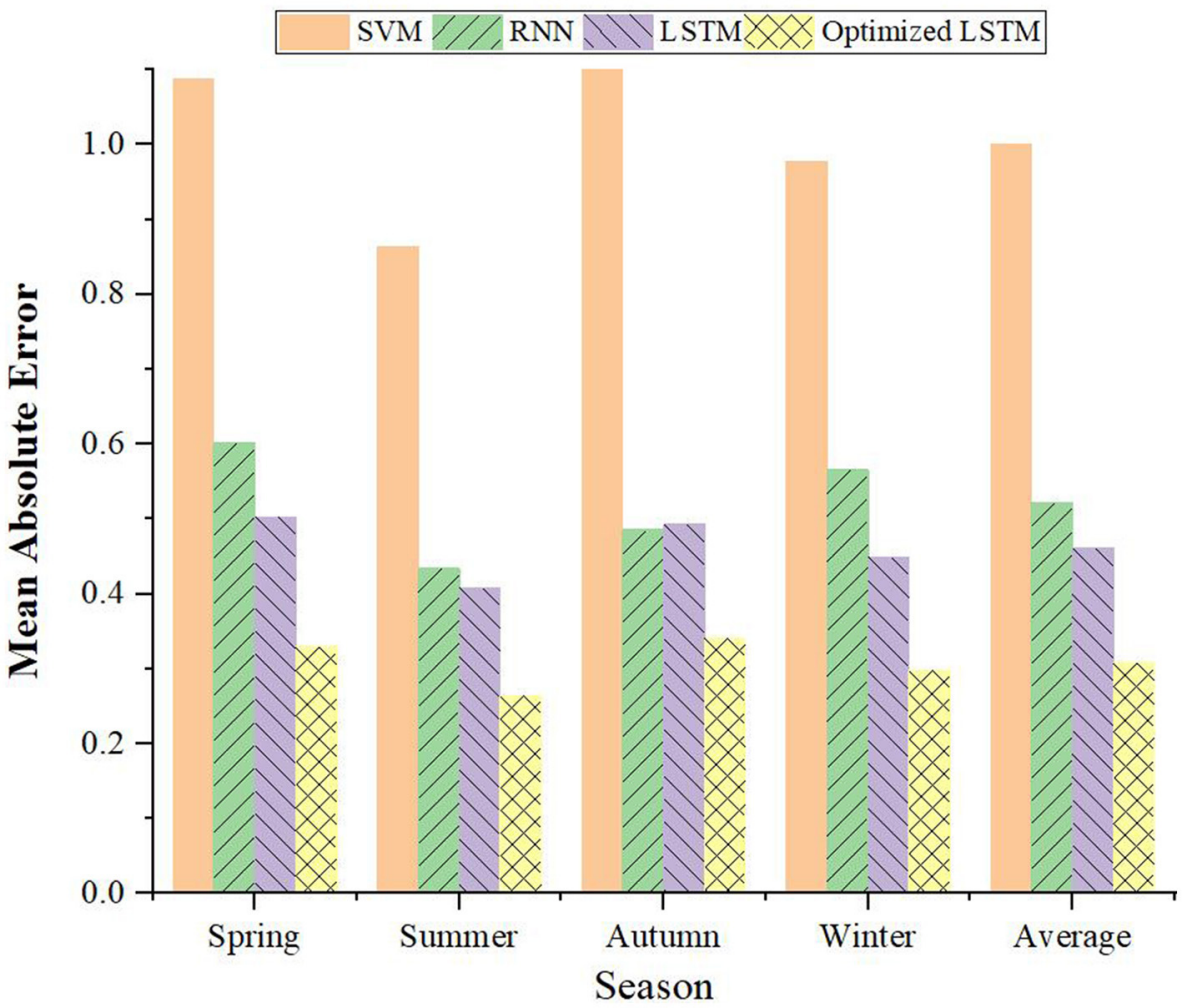
Comparison of load forecasting models for PDN in different seasons with a time scale of 1 h.

**Figure 11:**
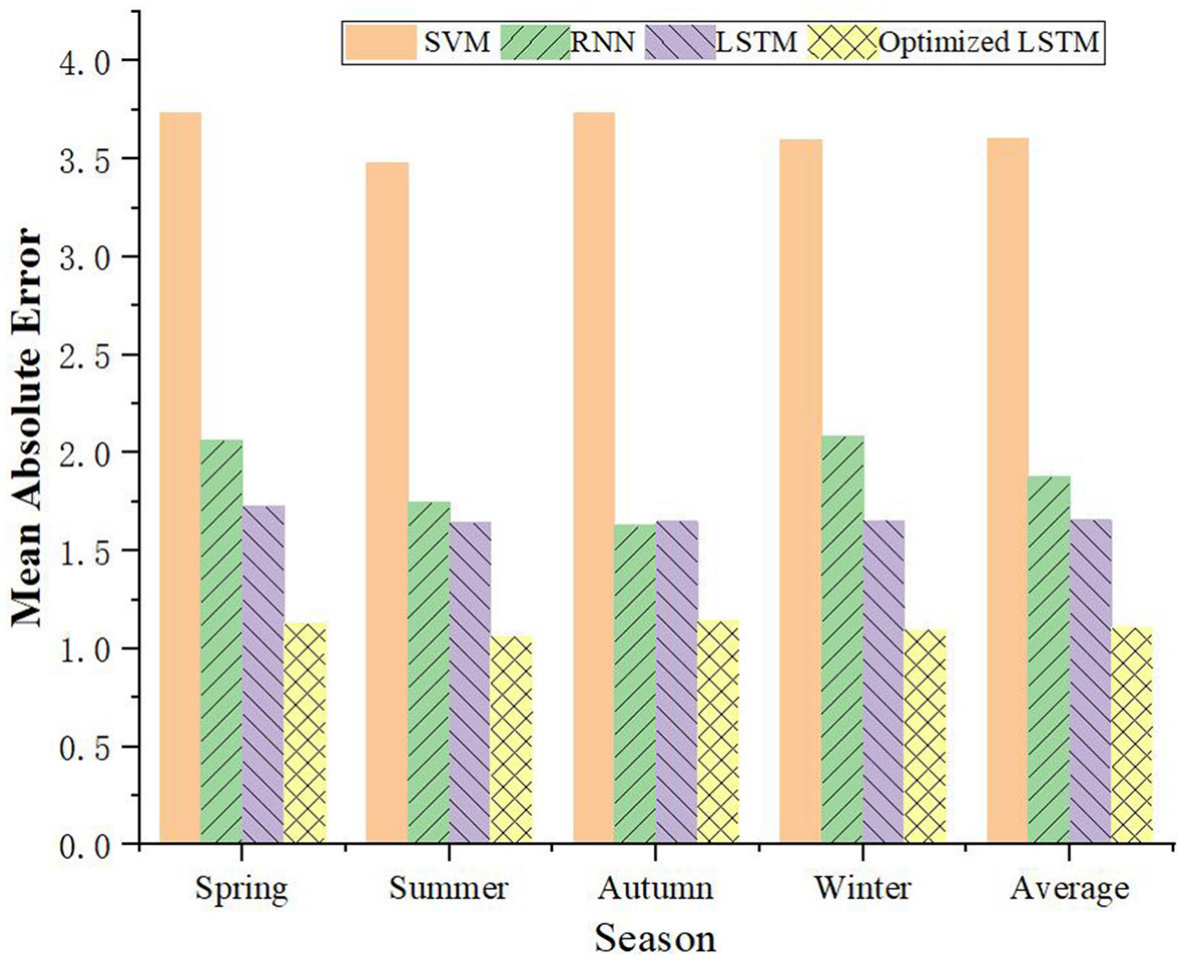
Comparison of load forecasting models for PDN in different seasons with a time scale of 4 h.

Taking the time scale of 1-h and 4-h as the basis, this study further verifies the voltage prediction of different PDN's load forecasting models in the four seasons, and the comparison results are portrayed in Figures 10, 11. It can be concluded that the prediction results of the improved LSTM model based on FDD are optimal in all seasons, especially as the prediction time scale increases. Taking summer with a time scale of 1 h as an example, the prediction MAE of the improved LSTM model is only 0.24, which reduces the prediction error of this model by about 35%. Even at a 4-h scale, the LSTM's MAE remains the lowest at 1.064 in summer. Therefore, the capability of the model in load forecasting of PV-PDN is further verified.

The proposed algorithm demonstrates significant practical value and effectiveness in the PV-PDN scenario. It can accurately predict voltage variations under different environmental conditions, and its prediction accuracy surpasses that of other models, especially as the prediction time scale increases. This capability provides strong support for the safe, reliable and efficient operation of PV power stations, helping maintenance personnel to promptly identify and resolve potential issues, thereby improving the operational efficiency and long-term stability of the PV power stations.

## 5 Conclusion

Driven by the rapid development of new power systems, the proportion of new energy is continuously increasing, and the scale of application and access rate of distributed PV in the low-voltage PDN are also steadily rising. The integration of distributed PV power generation, nonetheless, often exerts a substantial impact on the voltage distribution within PDN, giving rise to issues such as low voltage and voltage fluctuations. These issues severely impact the quality of daily life and production for users, further augmenting the uncertainty in grid operation and hindering the development of the social economy. Consequently, enhancing the state awareness capability of PDN is of paramount importance. Effective voltage prediction can provide data support for the safe and stable operation of PDN, thereby facilitating the resolution of voltage issues arising from the integration of distributed PV systems. In recent years, LSTM networks have demonstrated remarkable application potential in the realm of power load forecasting, and it offer a novel solution for PDN voltage prediction. Thus, a LSTM is extensively used in power load forecasting model of actual PDN based on DL and FDD is proposed in this study. By fast Fourier decomposition of the original quantity, the phase and amplitude of each frequency sine wave are acquired. Then LSTM is used to predict the low-frequency and high frequency components, respectively. The effectiveness of the proposed FDD-based LSTM model is verified by testing the historical operating data of PV-PDN. With the increase of the prediction time scale of the improved model, the error of the predicted results does not increase significantly. At a 1-h time scale, the MAE of the improved LSTM model is only 0.4554, much lower than that of other models. However, the proposed model requires a large amount of data for training and cannot be directly deployed on edge clients with limited computational resources for prediction. In the future, with the continuous development of edge computing and deep learning technologies, optimizing model computation efficiency to accommodate hardware constraints of edge devices and developing lightweight deep learning algorithms to reduce resource consumption, deploying prediction models at the edge side will become more feasible.

## Data availability statement

The dataset for this research was provided by a collaborating institution and contains sensitive information and usage restrictions. If other researchers need to obtain this dataset for further research or other reasonable requests, please contact the corresponding author.

## Author contributions

XZ: Conceptualization, Formal analysis, Investigation, Methodology, Validation, Writing – original draft, Writing – review & editing. JunlW: Conceptualization, Methodology, Validation, Writing – original draft, Writing – review & editing. JunW: Methodology, Resources, Supervision, Writing – original draft, Writing – review & editing. HW: Data curation, Validation, Writing – original draft, Writing – review & editing. LL: Conceptualization, Validation, Writing – original draft, Writing – review & editing.
